# Dual Immunomagnetic Nanobeads-Based Lateral Flow Test Strip for Simultaneous Quantitative Detection of Carcinoembryonic Antigen and Neuron Specific Enolase

**DOI:** 10.1038/srep42414

**Published:** 2017-02-10

**Authors:** Wenting Lu, Kan Wang, Kun Xiao, Weijian Qin, Yafei Hou, Hao Xu, Xinyu Yan, Yanrong Chen, Daxiang Cui, Jinghua He

**Affiliations:** 1 Outpatient Department, Zhujiang Hospital, Southern Medical University, 253 Gongye Road, Guangzhou, Guangdong 510280, China; 2 School of Electronic Information and Electrical Engineering, Shanghai Jiao Tong University, Shanghai 200240, China; 3 Shanghai Engineering Research Center for Intelligent Diagnosis and Treatment Instruments, Shanghai 200240, China; 4 School of Naval Architecture, Ocean & Civil Engineering, Shanghai Jiao Tong University, Shanghai 200240, China

## Abstract

A novel immunomagnetic nanobeads -based lateral flow test strip was developed for the simultaneous quantitative detection of neuron specific enolase (NSE) and carcinoembryonic antigen (CEA), which are sensitive and specific in the clinical diagnosis of small cell lung cancer. Using this nanoscale method, high saturation magnetization, carboxyl-modified magnetic nanobeads were successfully synthesized. To obtain the immunomagnetic probes, a covalent bioconjugation of the magnetic nanobeads with the antibody of NSE and CEA was carried out. The detection area contained test line 1 and test line 2 which captured the immune complexes sensitively and formed sandwich complexes. In this assay, cross-reactivity results were negative and both NSE and CEA were detected simultaneously with no obvious influence on each other. The magnetic signal intensity of the nitrocellulose membrane was measured by a magnetic assay reader. For quantitative analysis, the calculated limit of detection was 0.094 ng/mL for NSE and 0.045 ng/mL for CEA. One hundred thirty clinical samples were used to validate the test strip which exhibited high sensitivity and specificity. This dual lateral flow test strip not only provided an easy, rapid, simultaneous quantitative detection strategy for NSE and CEA, but may also be valuable in automated and portable diagnostic applications.

Carcinoma of the lungs, one of the most common malignant tumors worldwide, is the major cause of cancer-related deaths^1^. Most patients (more than 75%) are in middle–advanced stage when diagnosed, therefore, optimal therapeutic opportunities are unavailable^2^. Furthermore, the 5-year survival rate of patients with lung carcinoma is particularly low (about 15%). The poor prognosis of lung cancer is caused by lack of early diagnosis, thus screening to ensure early diagnosis is in urgent need of improvement. As most tumor markers are not specific to a particular tumor, it is inadequate to diagnose cancer using only a single marker. In order to improve lung cancer screening, it is necessary to simultaneously detect multiple tumor markers^3^. Small cell lung cancer, the most malignant subtype of lung cancer, develops very fast, metastasizes in the early stage, and the prognosis is very poor. In addition, small cell lung cancer is sensitive to radiation treatment and chemotherapy and if diagnosed at an early stage, and the median survival could be up to 24.1 months. However, only 5% of the patients are diagnosed early, therefore choosing effective tumor markers for early diagnosis is very important^4^. Neuron specific enolase (NSE), a glycolytic enzyme, is mainly found in neurons and peripheral nerve endocrine tissue, and is a sensitive and specific marker associated with small cell lung cancer^5^. The level of NSE in healthy individuals is <15.0 ng/mL and 60–81% of patients with small cell lung cancer have abnormally high levels^6^. At present, NSE is one of the most important markers for the diagnosis of small cell lung cancer. The positive serum NSE level in patients with small cell lung cancer was 72.4%, which was higher than that in patients with squamous cell carcinoma and adenocarcinoma (35.7% and 36.1%, respectively)^7^. Carcinoembryonic antigen (CEA) is a glycoprotein involved in cell adhesion, is normally generated during fetal development, and production stops before birth. CEA is used as a tumor marker in a variety of tumors, and plays an important role in the diagnosis and prognosis of lung cancer and 30% to 70% of patients with lung cancer have abnormally high levels of CEA^8^. When either NSE or CEA was detected, the sensitivity was relatively low and the specificity was poor for the diagnosis of small cell lung cancer. Thus, the detection of either serum NSE or serum CEA may not have obvious value for the early diagnosis of small cell lung cancer, and the simultaneous detection of then may improve the accuracy of lung cancer diagnosis^9^,^10^.

A variety of approaches have been proposed in the past few years to detect NSE and CEA. For example, electrochemical immunoassay^11^,^12^, chemiluminescence analysis^13^,^14^, fluorescence detection^15^,^16^, and enzyme-linked immunosorbent assay^17^. Most of these techniques require large and expensive instruments, complex operations, long analysis time and professional expertise, which results in poor application in early screening. Thus, there is a need to develop a simple, sensitive, low cost and rapid approach for the simultaneous detection of multiple tumor markers. The lateral flow immunoassay is a popular diagnostic method that can rapidly provide *in vitro* diagnostic results by non-trained personnel at a patient site. Due to its simplicity, speed, and no need for highly-qualified personnel, lateral flow immunoassay has been widely used in numerous fields, such as the detection of serum proteins (tumor markers, cardiac markers), pathogenic agents (bacteria, parasites, viruses), environmental pollutants, and drugs.

The lateral flow immunoassay usually utilizes colloidal gold, fluorescent material or magnetic nanoparticles as its label. Recently, a lateral flow immunoassay based on colloidal gold nanoparticles has attracted attention due to its simplicity, short analysis time and straightforward readout^18^. However, the limit of detection of the colloidal gold-based lateral flow test strip does not satisfy the requirements and only provides qualitative or semi-quantitative analysis due to simple equipment and naked eye observations. Nowadays, optical detection of the intensity of fluorescence labels such as quantum dots^19^, near infrared dyes^18^,^20^, gold nanoclusters^21^,^22^ and upconversion nanomaterials^23^, have been widely used in lateral flow immunoassay. During the detection, optical signals can be influenced by specular reflection, scattering, self-absorption, and the fluorescence signal can be quenched, which is unfavorable for batch production and preservation. In addition, the fluorescence probes require complex purification/separation procedures, which may limit their large-scale application in lateral flow immunoassay. Moreover, at most 10 μm at the top of the nitrocellulose membrane, which is a few hundred micrometers thick, is measured by a signal reader or the naked eye, thus effective information in the test line cannot be obtained.

Due to the absence of a magnetic background and photo-bleaching in analyte samples, the lateral flow test strip based on magnetic nanoparticles exhibit a high “signal-to-noise” ratio and maintain a stable signal compared to the color-based or optical-based lateral flow test strip^24^. The lateral flow immunoassay based on magnetic nanoparticles can be manipulated with an external magnetic field for magnetic separation, which can enhance the efficiency and diminish the assay time^25^. The lateral flow immunoassay provides quantitative results by measuring the magnetic signal of the test zone using a magnetic assay reader^26^, which improves the sensitivity and provides a better means of quantification^27^. Based on the above advantages, it is possible to use lateral flow test strip based on magnetic nanoparticles for the detection of NSE and CEA. This type of study has not been reported previously.

In this study, a kind of carboxyl-modified(COOH–) magnetic nanobeads with high saturation magnetization were used as a probe to establish an immunomagnetic nanobeads-based lateral flow test strip for the simultaneous detection of NSE and CEA. This immunoassay system not only accurately analyzed NSE and CEA separately, but also had no obvious influence on NSE and CEA when detected simultaneously. This technique exhibited satisfactory results when clinical serum samples were used to evaluate the sensitivity and specificity of this test strip and the results showed good agreement with those using a commercial electrochemiluminescent immunoassay kit. This novel lateral flow test strip provides an easy, rapid simultaneous quantitative detection strategy for NSE and CEA in serum, and is suitable for development in point-of-care testing.

## Results and Discussion

### Principle of the method

**Figure 1:**
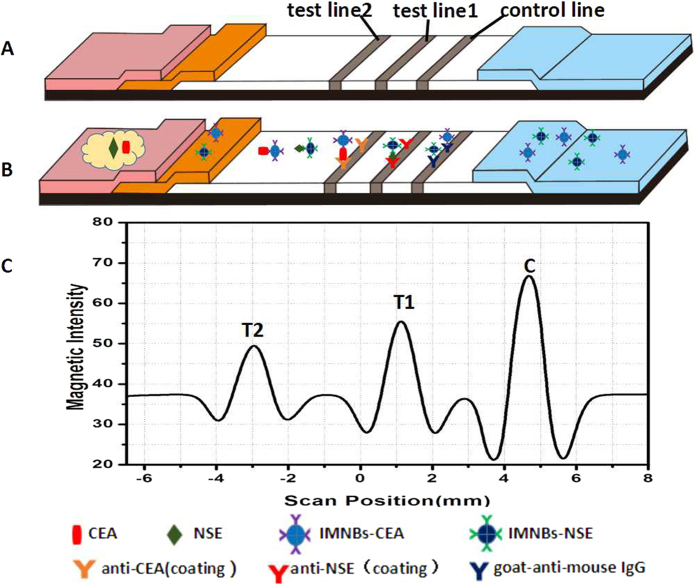
Schematic illustration of the lateral flow assay. (**A**) Components of test strip. (**B**) Samples with NSE and CEA were applied to the sample pads, and migrated along the strip, NSE and CEA combined with their corresponding probe respectively. The complexes migrated along the membrane and were captured by the coating antibodies to form a sandwich complexes on T1 and T2. Then the excess probes were captured by control line. (**C**) The intensity of magnetic signal was measured by a magnetic assay reader. The peak values from right to left represent the magnetic intensity of control line, test line 1 and test line 2, respectively.

The principle of the immunomagnetic nanobeads-based lateral flow test strip was based on the antigen–antibody reaction to form a sandwich format. As illustrated in Fig. 1, the antibodies of NSE and CEA were immobilized on the nitrocellulose membrane as test line 1 and test line 2. When the liquid sample migrated to the conjugate pad, the immunomagnetic probes of NSE and CEA dissolved and bounded with the target analytes. The formed complexes migrated to the membrane and were captured by test lines. The residual conjugates continued to migrate along the membrane and were captured by the control line, which served as the internal control. In contrast assay, PBS with no analyte was used as the control. If the control line could not be seen, the strip was identified invalid. After appropriate immunoreaction time, a cartridge packaging test strip was inserted into the magnetic assay reader to detect the magnetic signal of the T lines and C line. Data were reported as the intensity of the magnetic signal which was proportional to the amount of immune complexes formed in the T lines or C line, as show in Fig. 1C. To quantitatively analyze NSE and CEA, the ratio of the intensity of T/C was used for measurement to offset the background and inherent heterogeneity of the test strip. On the basis of the above, standard curves were plotted using the proportional relationship between the concentration of analytes and the T/C.

### Characterization of the immunomagnetic probes

**Figure 2:**
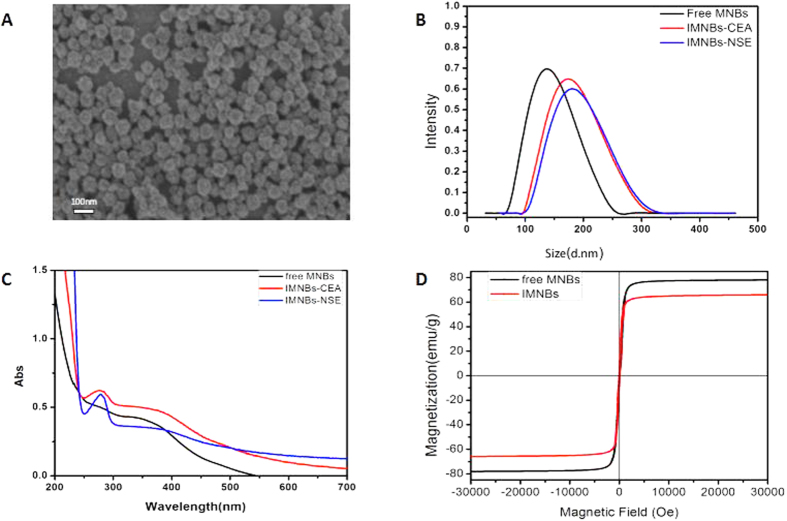
Characterization of free magnetic nanobeads and magnetic probes. (**A**) Transmission electron microscopy image of magnetic nanobeads. (**B**) Hydrodynamic size distribution of free magnetic nanobeads and magnetic probes. (**C**) UV-Vis absorption spectroscopy of free magnetic nanobeads and magnetic probes. **(D**) Magnetization curves of free magnetic nanobeads and magnetic probes.

The magnetic nanobeads were synthesized in our laboratory, and the morphology of the final product was characterized with transmission electron microscopy. A particle sizing system was used to determine their hydrodynamic diameter and zeta potential, and the Quantum Design PPMS-9 T (EC-II) system was used to determine their magnetic properties. Transmission electron microscopy revealed that magnetic nanobeads were monodispersed, spherical in shape, and had an average diameter of 80 nm (Fig. 2A). The hydrodynamic size distribution and zeta potential of the magnetic nanobeads before and after labeling with antibody were measured using a particle sizing system. As shown in Fig. 2B, due to the thickness of the hydrated layer on the nanobeads surface, the hydrodynamic size was much larger than that displayed by transmission electron microscopy. The hydrodynamic size of free nanobeads was 150 nm, and the diameter increased to 175 nm and 168 nm after conjugation with anti-NSE monoclonal antibody and anti-CEA monoclonal antibody, respectively, and there was no aggregation during the coupling reaction. Because of the abundant carboxyl groups on their surfaces, the magnetic nanobeads had a negative surface charge. The zeta potential is a key indicator of the stability of the system dispersion, and a high zeta potential (±30 mV) will confer stability. The average zeta potential of free nanobeads was approximately −27.58 ± 0.23 mV, and changed to −20.5 ± 0.19 mV and −22.4 ± 0.22 mV after conjugation with antibody of NSE and CEA, respectively. The high level of surface potential ensured that the nanobeads were monodispersed during electrostatic repulsion. In order to confirm the binding of the magnetic nanobeads and antibodies, ultraviolet absorption spectra of magnetic nanobeads before and after coupling were detected using an ultraviolet spectrophotometer. As shown in Fig. 2C, the free nanobeads has no absorption peak when scanned at a wavelength range of 200–800 nm. We know that the absorption peak of the pure protein was located at 280 nm. The spectrum of the magnetic probes had an absorption peak at 270 nm after conjugation with antibodies, indicating the presence of an antibody coating, and the protein absorption peak showed a slight hypsochromic shift after coupling to the nanobeads (from 280 nm to 270 nm).

As shown in Fig. 2D, the magnetization curves show that the saturation magnetization of free magnetic nanobeads was 80 emu/g, and maintained a high magnetism value of 67 emu/g after conjugation with the antibody. When a magnetic scaffold was used to separate the magnetic nanobeads, the magnetic nanobeads totally aggregated within 30 s. This magnetic property indicates that the magnetic nanobeads had a strong magnetic responsivity, which is an advantage in magnetic separation and is easily recovered after an external magnetic field is applied. The aforementioned characterization indicates that the antibodies had combined with the magnetic nanobeads and that the immunomagnetic probes did not aggregate during coupling and were stably dispersed in solution.

### Optimization of experimental parameters

#### Choice of magnetic nanobeads size

**Figure 3:**
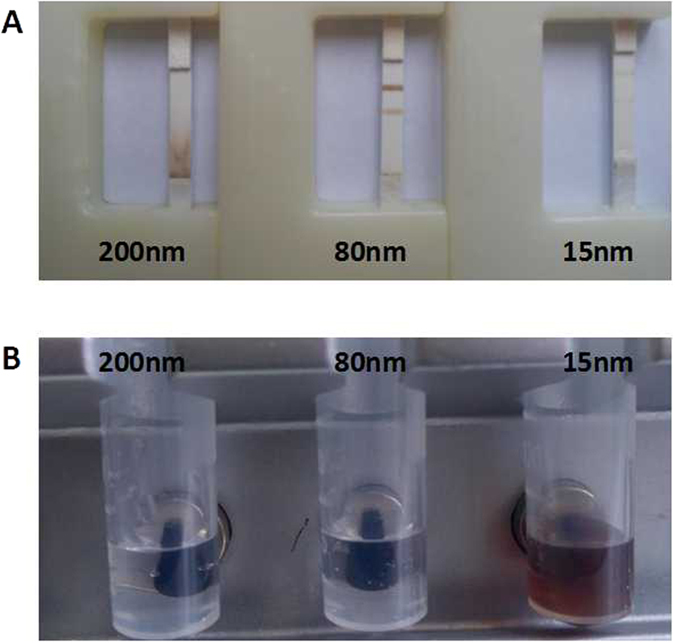
Different sizes of magnetic nanobeads (200 nm, 80 nm, and 15 nm). (**A**) Magnetic nanobeads with different sizes, distribution along the test strip. (**B**) The degree of aggregation of magnetic nanobeads with different sizes using the magnetic scaffold.

In order to improve the assay performance, a series of optimization steps were carried out. Firstly, three types of magnetic nanobeads of different sizes (15 nm, 80 nm and 200 nm) were chosen to optimize assay performance. As shown in Fig. 3A, the best results were obtained with 80 nm nanobeads, with obvious signals at both the test line and the control line. There was a substantial signal at the border between the conjugate pad and the nitrocellulose membrane when 200 nm particles were used, probably because the 200 nm particles were too big to migrate via the nitrocellulose membrane. When the 15 nm particles were used, the signal at the T line was not observed in the positive samples, because the capillary speed was too fast, and there was not enough time for the antigen-antibody binding reaction. In addition, when the magnetic scaffold was used to separate the magnetic nanobeads, the degree of aggregation of 15 nm magnetic nanobeads was still very poor after 30 min, and when 80 nm or 200 nm particles were used, total aggregation was observed within 30 s (Fig. 3B). This may have been because the force exerted by the magnetic field was related to the diameter of the magnetic nanobeads and had a third-power relationship with the diameter^28^. Thus, 15 nm particles require more time for separation by the same magnetic field. We also tried to separate the 15 nm particles by centrifugation, but the separation efficiency was very poor, and a large number of particles were lost. Therefore, the 80 nm magnetic nanobeads were considered optimum for the lateral flow immunoassay.

#### Volume of the standard antigen

In order to ensure smooth performance of the lateral flow test and the consumption of serum was minimum, the volume of the standard antigen (50 ng/mL) was investigated by adding 40, 60, 80, and 100 μL to the sample pad, respectively. When 100 μL of solution was added, the immunomagnetic probes were stranded on the nitrocellulose membrane and did not migrate towards the absorbent pad, as the amount of liquid was greater than the absorption ability. A sample volume of 40 μL was subsequently investigated. The magnetic probes were not successfully released from the conjugate pad, resulting in weak signals of both T lines and the C line. Thus, the 40 μL volume may have been too small to dissolve the probes and migrate to the nitrocellulose membrane. When the 60 μL and 80 μL volumes were used for detection, the magnetic probes migrated along the nitrocellulose membrane smoothly, and both T lines and the C line showed strong signals. Therefore, the 60 μL volume was chosen as the optimal sample volume for subsequent experiments based on the above results.

#### Optimum amount of added antibody

**Figure 4:**
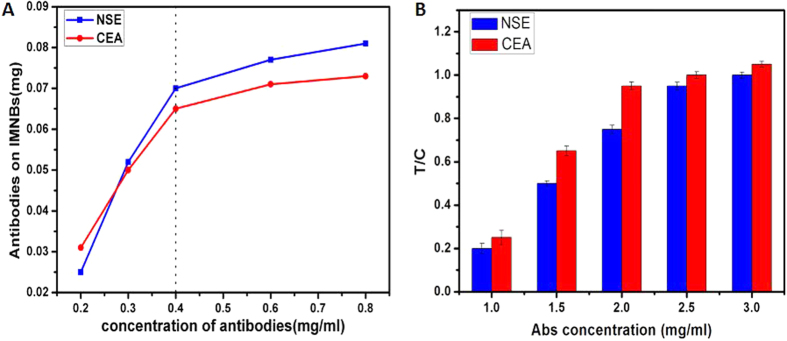
(**A**) Relationship between the amount of antibody on the magnetic probes and the amount of antibody participating in coupling. (**B**) Relationship between the T/C ratio and concentration of antibody at the test lines.

The BCA protein assay kit was used to calculate the immobilization efficiency. During this procedure, different concentrations of the standard protein was used to construct a standard curve. The standard curve was y = 140.51x − 1.5098 (R^2^ = 0.99531), where y is the protein absorbance and x is the protein concentration. From the standard curve, we calculated the residual antibody in the supernatant liquid, and the amount of antibody coating the magnetic probe could be calculated indirectly. The improvement in the immobilization efficiency improved the sensitivity of this test, which has been verified in our previous experiments. To improve the immobilization efficiency, and save on the cost of antibodies at the same time, the amount of antibodies added to the coupling reaction was optimized. Figure 4A shows the relationship between the amount of antibodies on magnetic probes and the amount of antibody added to the reaction tube. The amount of antibodies on magnetic probes increased when the amount of antibody added to the reaction tube increased. However, when the antibodies in the reaction tube varied between 0.4–0.8 mg/mL, the increase in the antibodies on the probes was not statistically significant (*P  *> 0.05). Therefore, we chose 0.4 mg/mL as the optimal concentration of antibody for the coupling reaction. When the optimal concentration of antibody was 0.4 mg/mL, the amount of antibody coating the magnetic nanobeads was 70 μg for NSE, and 65 μg for CEA.

#### The amount and ratio of NSE probes and CEA probes on the conjugate pad

The intensity of the magnetic signal depends on the amount of magnetic probes captured by the test lines, thus the amount and ratio of NSE probes and CEA probes on the conjugate pad would affect the signal of the T lines and should be optimized. In this part, a volume of 60 μL was used, and the analyte concentration was 50 ng/mL for both NSE and CEA. The prepared probes of NSE and CEA were mixed at different concentration ratios and then applied to the conjugate pad to fabricate a series of test strip. We found that the magnetic signals were strongest both for NSE and CEA, when the concentration ratio of NSE probes and CEA probes was 1.2:1.0, and the amount of probes was 10 μL. According to the results of previous experiments, we found that the antigen–antibody combining ability of NSE was weaker than that of CEA. Therefore, the amount of NSE probe was slightly greater than that of CEA probe. We also discovered that both too many and too few magnetic probes could degrade the accuracy of quantitation. If there are too few probes on the conjugate pad, most of the analytes in the sample would not be involved in the immunoreaction, resulting in a decrease in detection sensitivity. However, a large number of probes will result in a high magnetic background leading to poor quantitative detection, because the excess probes are distributed on the membrane uniformly and impact the signal of the test lines and control line.

#### The amount of anti-NSE (coating) and anti-CEA (coating) on the test lines

To increase the sensitivity of the lateral flow test strip and to decrease the cost of reagents, the amount of antibody on the test lines was optimized by depositing different concentrations (1.0, 1.5, 2.0, 2.5 and 3.0 mg/mL) of antibody onto the test lines. For this assay, the optimal sample volume of 60 μL was used, and the analyte concentration was 50 ng/mL both for NSE and CEA. The relationships between the magnetic intensity T/C and concentration of antibodies at the test lines are shown in Fig. 4B. With an increase in the antibody concentration, the T/C ratio increased. However, when the concentrations increased from 2.5 to 3.0 mg/mL for NSE and from 2.0 to 3.0 mg/mL for CEA, the increase in the magnetic intensity T/C was not significant (*P* > 0.05). Therefore, the optimal antibody concentration was 2.5 mg/mL for NSE and 2.0 mg/mL for CEA considering the reagent consumption.

#### Quantification of CEA and NSE using the magnetic lateral flow test strip

**Figure 5:**
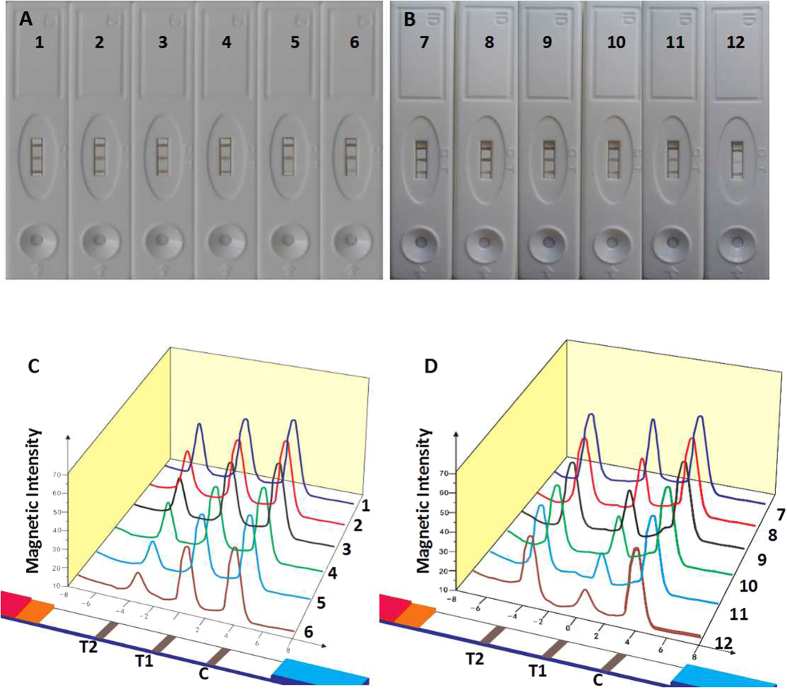
Simultaneous detection of NSE (T1) and CEA (T2). (**A**) The color changed of the T2 obtained by the naked eye. From 1–6, the concentration of CEA changed from 50 ng/ml to 1 ng/ml, while NSE is 50 ng/ml. (**C**) The magnetic signal recorded by the magnetic assay reader. (**B**) The color changed of the T1 obtained by the naked eye. From 7–12, the concentration of NSE changed from 50 ng/ml to 1 ng/ml, while CEA is 50 ng/ml. (**D**) The magnetic signal recorded by the magnetic assay reader.

**Figure 6:**
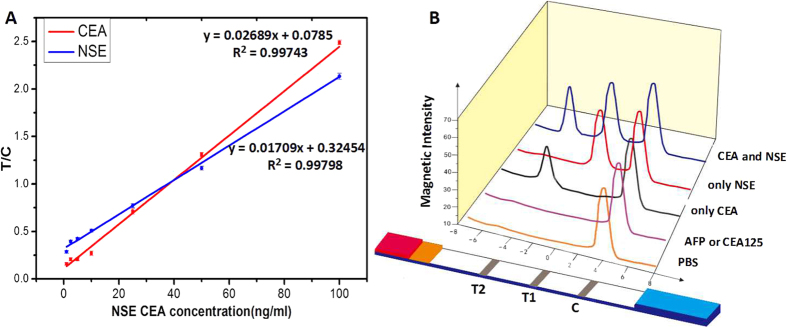
(**A**) Standard curve for quantitative detection of NSE and CEA. (**B**) Cross-reactivity of the test strip. AFP and CA125 were used as interferential antigens and PBS was used for blank control.

Following successful fabrication of the lateral flow test strip, we used the strip for the simultaneous quantification of NSE and CEA. The standard curves of NSE and CEA were constructed based on the measurement of different concentrations of analyte standards (1.0, 2.5, 5.0, 10.0, 25.0, 50.0 and 100.0 ng/mL), which were prepared in standard dilution buffer. When different concentrations of the antigen were detected, there was a brown band appeared on test line, and the color of the band gradually intensified as the antigen concentration increased. Therefore, it provides a qualitative or semiquantitative measure visible to the naked eye. The limit of detection by the naked eye was 1.0 ng/mL for NSE and 0.5 ng/mL for CEA. The color changes in the T lines at different concentrations were not obvious to the naked eye (Fig. 5A and B), but when the magnetic signal was recorded with a magnetic assay reader, the signal gradually intensified with an increase in concentration (Fig. 5C and D). The magnetic assay reader uses a C-shaped electromagnet to magnetize magnetic probes. During the measurement, this magnetization is measured with an array of thin-film induction coils. Because the magnetization is proportional to the amount of probe trapped in the test zone, the magnetic assay reader can calculate the concentration of the analyte and provide a quantitative value for the assay. The magnetic strength at different positions on the nitrocellulose membrane is shown as the magnetic scan profile. After recording the magnetic signal, the standard curve was obtained by plotting the linearity of T/C against the concentration of NSE or CEA. As represented by the equation in Fig. 6A, the linear equation for NSE was y = 0.01709x + 0.32454 (R^2^ = 0.99798), and for CEA was y = 0.02689x + 0.0785 (R^2^ = 0.99743). The linear correlation coefficients (R^2^) were relatively high, allowing the easy determination of the NSE and CEA concentrations in an unknown serum sample. The limit of detection was calculated to be 0.094 ng/mL for NSE and 0.045 ng/mL for CEA. The coefficients of variation were all <10% (n = 5) at each concentration of both CEA and NSE, which showed an acceptable level of reproducibility for simultaneous quantification of NSE and CEA.

#### Evaluation of cross-reactivity

The specificity of the antibody is an important factor in immune detection. In order to estimate the specificity of the lateral flow test strip, cross-reactivity tests between the target analytes and the interference (AFP and CA125, two of the most common tumor markers in clinical diagnosis) were performed. As shown in Fig. 6B, when the assay was conducted with only one antigen (NSE or CEA), only one test line (T1 or T2) appeared. When detecting AFP or CA125, neither test line 1 nor test line 2 was observed, this was similar to when PBS was used as the blank control. We then used the serum to verify the cross-reaction, and the results were consistent with those using standard antigens. These negligible cross-reactivity results demonstrated that the effect of interference was insignificant, and both NSE and CEA could be detected simultaneously with no obvious influence on each other.

#### Detection in clinical human serum samples

To evaluate the potential of the proposed method for clinical application, 130 clinical samples (100 positive and 30 negative) were quantitative analyzed by the proposed test strip, and accurate values of the analytes could be obtained. The actual detection limits for serum were 0.75 ng/mL and 0.40 ng/mL for NSE and CEA, respectively. Accurate values for NSE and CEA in clinical serum also were obtained with an electrochemiluminescence immunoassay kit, and the serum levels of 15.0 ng/mL for NSE and 5.0 ng/mL for CEA were regarded as the positive thresholds for a clinical diagnoses^29^. As shown in Table 1, this method had a sensitivity of 97% and 95% for NSE and CEA, and a specificity of 87% and 93%, respectively. In order to compared the quantitative results between the lateral flow test strip and the commercial electrochemiluminescence immunoassay kit, the results of 30 serum samples were chosen randomly to confirm the consistency. Good agreement between the two methods could be observed, and the correlation (n = 30, R^2^ = 0.9926, *P* < 0.001) of NSE and (n = 30, R^2^ = 0.9922, *P* < 0.001) of CEA values were considered excellent (Supplementary Fig. 1). A series of negative human serum samples were spiked with different concentrations (5.0, 25.0, 50.0 ng/mL) of NSE and CEA in a spiked recovery experiment to evaluate the accuracy of this assay. As shown in Table 2, the recovery rates for the spiked NSE and CEA samples were 94.4%–106.0% and 96%–104.8%, respectively (n = 5). The relative standard deviations were 2.7%–5.9% for NSE and 2.5%–5.2% for CEA. These results indicated that the lateral flow test strip exhibited high accuracy and can be used in clinical analysis.

## Conclusions

In this study, a dual immumomagnetic nanobeads-based lateral flow test strip was successfully constructed and validated which simultaneously and quantitatively detected NSE and CEA. This novel analysis platform has the advantage of speed, low cost and high sensitivity for simultaneous analysis. Under optimal conditions, the limits of detection for the quantitative detection of NSE and CEA were as low as 0.094 ng/mL and 0.045 ng/mL, respectively. The results of cross-reactivity tests showed that interference had insignificant effects on the analytes, and NSE and CEA were simultaneously detected without influencing each other. The accuracy of the lateral flow test strip validated using spiked human serum met the requirements for quantitative analysis, and the results using clinical samples also demonstrated that the lateral flow test strip was reliable for measuring multiple tumor markers. According to the aforementioned results, this assay would be helpful in the early diagnosis of lung cancer in medical institutions, and may be a good choice for point of care testing. Future work will further extend the capability of this method by simultaneously detecting additional multiple tumor markers on individual test lines.

## Material and Methods

### Chemicals and equipment

The carboxyl-modified (COOH–) magnetic nanobeads (80 nm) were synthesized in our laboratory. The goat anti-mouse IgG antibody, NSE corresponding monoclonal antibodies (L1C00501 and L1C0050503), mouse anti-CEA monoclonal antibodies (L1C00205 and L1C00202), and standard antigens (NSE, CEA, AFP and CA125) were all purchased from Shanghai Linc-Bio Science Co., Ltd., Shanghai, China. Bovine serum albumin (BSA), 2-(N-morpholino)ethanesulfonic acid (MES), 1-ethyl-3-(3-dimethyllaminopropyl)-carbodiimide hydrochloride (EDC) and N-hydroxy-succinimide (NHS) were obtained from Aladdin (Los Angeles, CA, USA). Deionized water was used in the experiments. Other reagents and solvents were of analytical grade, and the nitrocellulose membrane (Millipore 75, Millipore 90) were provided by Jiening Biological Technology Co., Ltd., Shanghai, China. The conjugate pad (glass fiber membrane, Ahlstrom 8964), sample pad (glass fiber membrane, GL-b01), adsorbent pad, and the polyvinyl chloride plate were supplied by Shanghai JieYi Biotechnology Co., Ltd., Shanghai, China. The magnetic assay reader was purchased from Magna Bioscience LLC (San Diego, CA, USA). The magnetic separation rack was from Hon-Pro Technology Development Co., Ltd., Shenzhen, China, and the XYZ dispensing system (XYZ3060) was purchased from BioDot Inc., Irvine, CA, USA. The vortex mixer and incubator were commonly used equipment in the laboratory. The clinical serum samples were from Zhujiang Hospital, Southern Medical University, Guangzhou, China.

### Preparation of magnetic nanobeads

Water soluble magnetic nanobeads with carboxyl groups were synthesized using a modified one-step hydrothermal synthesis method^30^. Briefly, citrate as a surface ligand was dissolved to form a clear solution, and then vitamin C as the reducing agent was added immediately after the addition of ferrous sulfate. The mixture was sealed, placed in an autoclave and heated at 200 °C for 10 h. When the product had cooled to room temperature, a purification step was performed by magnetic separation. The powder product was then collected by vacuum drying. Morphology of the final product was characterized by transmission electron microscopy, a particle sizing system was used to determine hydrodynamic diameter and zeta potential, and a Quantum Design PPMS-9 T (EC-II) system was used to determine magnetic properties.

### Preparation of the immunomagnetic probes for CEA and NSE

The antibodies were immobilized with the classical EDC/NHS method. One milligram of magnetic nanobeads was suspended in 250 μL of activated buffer (0.01 M MES, pH = 5.5). Then, 2.5 mg of EDC and 2.5 mg of NHS were added to the solution. Following activation for 30 min, the excess EDC and NHS were removed via magnetic separation. Then 250 μL of conjugating buffer (pH = 9.0, 0.005 M) was added to re-suspend the activated magnetic nanobeads. Subsequently, 0.1 mg antibody of NSE (L1C00505) or antibody of CEA (L1C00202) was added to the activated magnetic nanobeads solution. The mixture was incubated in a horizontal shaker for 3 h at 37 °С. Following a magnetic washing step, 250 μL blocking buffer (containing 10% BSA) was added and reacted for 1 h at room temperature. The probes were magnetically washed twice with BST buffer (pH 8.0), and re-suspended in 500 μL of BST and stored at 4 °C for further use.

### Fabrication of magnetic lateral flow test strip

The magnetic lateral flow test strip was composed of a sample pad, conjugate pad, nitrocellulose membrane, and absorbent pad. The conjugate pad was immersed in treatment buffer containing 5% sucrose and 2% trehalose, and the sample pad was treated with treatment buffer containing 2% NaCl, 0.5% BSA, and 0.1% polyvinylpyrrolidone, and then dried at 37 °C overnight. The prepared probes for CEA and NSE were mixed at the desired ratio and dispensed onto the conjugate pad, then dried at 37 °C overnight and stored at 4 °C. To prepare the test lines and control line, 2.5 mg/mL solution of NSE antibody (L1C00501) and 2 mg/mL solution of CEA antibody (L1C00205) were immobilized on the nitrocellulose membrane as test line 1 and test line 2. Goat anti-mouse IgG at the concentration of 1 mg/mL was coated as the control line. After immobilization, the nitrocellulose membrane was dried at 37 °C in the oven for 1 h. All the components of the test strip were attached onto a polyvinyl chloride plate with appropriate overlaps (usually 1–2 mm) to ensure that the sample solution could migrate through the whole test strip. The assembled strip was then cut to a width of 3 mm and length of 80 mm using an automated cutter and stored in a dry sealed box at 4 °C for further use.

### Assay procedure

To perform the magnetic assay, the prepared test strip was placed on a clean horizontal platform, 60 μL sample solution containing NSE and CEA was dropped onto the sample pad and migrated along the strip under capillary action. PBS without NSE and CEA was used as control and each sample test was repeated for 3 times under the same condition. After appropriate immunoreaction time, the test strip was placed into the magnetic assay reader, followed by recording magnetic intensity of the test lines and control line to quantify the analytes.

### Clinical samples

The study was approved by the Medical Ethics Committee of the Southern Medical University, Guangzhou, China. All patients signed an informed consent and all methods were performed in accordance with the relevant guidelines and regulations. A total of 130 serum samples taken from patients were collected from Zhujiang Hospital, Southern Medical University, including 100 positive and 30 negative serum samples. All samples were stored at −80 °C until analyzed. Accurate values for NSE and CEA were obtained with a commercial electrochemiluminescence immunoassay kit.

### Statistical analysis

For each experiment, at least three replicates were performed, and the data were presented as mean ± standard deviation. Statistical consistency was evaluated with a consistency check. Statistical significance was set at *P* < 0.01. All data were analyzed by SPSS 21.0, and diagrams were generated with Origin Pro 8.0.

## Additional Information

**How to cite this article:** Lu, W. *et al*. Dual Immunomagnetic Nanobeads-Based Lateral Flow Test Strip for Simultaneous Quantitative Detection of Carcinoembryonic Antigen and Neuron Specific Enolase. *Sci. Rep.* **7**, 42414; doi: 10.1038/srep42414 (2017).

**Publisher's note:** Springer Nature remains neutral with regard to jurisdictional claims in published maps and institutional affiliations.

## Supplementary Material

Supplementary Information

## Figures and Tables

**Table 1:** Clinical test of CEA and NSE by the immunomagnetic nanobeads-based lateral flow test strip.

Sample Size	Tested (+)		Tested (−)			Validity		*Kappa*		*P*	
	NSE	CEA	NSE	CEA		NSE	CEA	NSE	CEA	NSE	CEA
100 Positive	97	95	3	5	sensitivity	97%	95%	0.847	0.853	0.001	0.001
30 Negative	4	2	26	28	specificity	87%	93%				

**Table 2:** Recovery of CEA and NSE by the immunomagnetic nanobeads-based lateral flow test strip (n = 5).

Sample	CEA			NSE		
Added(ng/ml)	50	25	5	50	25	5
Found(ng/ml)	52.4	24.5	4.8	53	23.6	4.9
Recovery(%)	104.8	98	96	106	94.4	98
RSD(%)	2.5	4.1	5.2	5.9	2.7	3.4
