# Supplementary material for: Dual Immunomagnetic Nanobeads-Based Lateral Flow Test Strip for Simultaneous Quantitative Detection of Carcinoembryonic Antigen and Neuron Specific Enolase
Source: Sci Rep. 2017 Feb 10;7:42414. doi: 10.1038/srep42414 (PMC5301198; doi:10.1038/srep42414)
Supplement: Supplementary Information [file srep42414-s1.doc]

**Supplementary Information**

**Dual Immunomagnetic Nanobeads-Based Lateral Flow Test Strip for Simultaneous Quantitative Detection of Carcinoembryonic Antigen and Neuron Specific Enolase**

Wenting Lu^1^, Kan Wang^2,3^*, Kun Xiao^2^, Weijian Qin^2^, Yafei Hou^2^, Hao Xu^4^**,** Xinyu Yan^1^,Yanrong Chen^1^, Daxiang Cui^2,3^,Jinghua He^1＊^

^1^Outpatient Department, Zhujiang Hospital, Southern Medical University, 253 Gongye Road, Guangzhou, Guangdong 510280, China.

^2^ School of Electronic Information and Electrical Engineering, Shanghai Jiao Tong University, Shanghai 200240, China.

^3^Shanghai Engineering Research Center for Intelligent Diagnosis and Treatment Instruments, Shanghai 200240, China.

^4^ School of Naval Architecture, Ocean & Civil Engineering, Shanghai Jiao Tong University, Shanghai 200240, China

**Corresponding Author:**

Kan Wang and Jinghua He

*Corresponding Email address: KanWang (wk_xa@163.com)

or Jinghua He [(hjh5258@foxmail.com)](mailto:(hjh5258@foxmail.com))

**Supplementary Information: 3 pages**

**Quantitative information with respect to correlation between the lateral flow assay and the commercial electrochemiluminescent kit.**

To demonstrate the clinical application of the test strip, a total of 130 serum samples were analyzed by the proposed method. And the results of 30 serum samples were chosen randomly to compared with those obtained by an electrochemoluminescence kit. As shown in Supplementary Fig.1, good agreement between two methods was observed; the correlation (n= 30, r ^2^=0.9926, p < 0.001) of NSE and (n= 30, r ^2^=0.9922, p < 0.001) of CEA values were considered excellent. Thus, the results confirm that the immunomagnetic nanobeads-based lateral flow test strip offers excellent performance for the determination of NSE and CEA in human serum in a clinical setting.


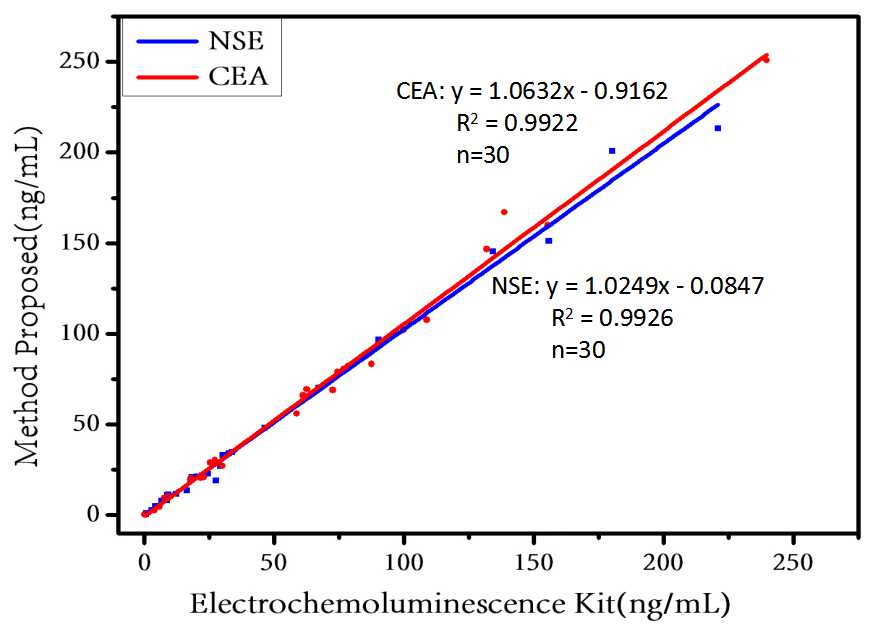


**Supplementary figure 1**. Compare the quantitative results of NSE and CEA in 30 serum with the proposed method and the electrochemoluminescence kit.
